# Mendelian Randomization Analysis of the Possible Causal Relationships Between Neurodevelopment‐Related Proteins and Bipolar Disorder

**DOI:** 10.1002/brb3.70442

**Published:** 2025-03-23

**Authors:** Yanyan Li, Qianqian Gui, Shurong Ren, Zhifen Liu, Aixia Zhang, Penghong Liu, Xueping Zhou, Ning Sun, Chunxia Yang

**Affiliations:** ^1^ Shanxi Medical University Taiyuan China; ^2^ Department of Psychiatry First Hospital of Shanxi Medical University Taiyuan China

**Keywords:** bipolar disorder, ITIH5, Mendelian randomization, neurodevelopment, NFASC

## Abstract

**Background:**

Bipolar disorder (BD) is a complex mental condition of which the mechanism of onset remains unclear. Mendelian randomization (MR) allows evaluation of the causal effects of biomarkers by minimizing the risks of reverse causation and confounding factors. In this study, MR was used to assess the causal relationships between neurodevelopment‐related proteins and BD, thereby providing potential evidence for the neurodevelopmental hypothesis of this mental disorder.

**Methods:**

Leveraging data from large‐scale genome‐wide association studies (GWASs), the associations between six neurodevelopment‐related proteins and BD were analyzed using five MR approaches; namely, inverse‐variance weighted, weighted median, MR–Egger, simple mode, and weighted mode methods. The neurodevelopment‐related proteins were selected in the study with 5368 European descents. GWAS of BD come from the Psychiatric Genomics Consortium (*N*_Case_ = 41,917, *N*_Control_ = 371,549).

**Results:**

The analyses identified robust causal relationships between BD and the proteins inter‐alpha‐trypsin inhibitor heavy chain (ITIH)5 (OR = 1.08, 95% CI = 1.00–1.17, *p* = 0.04) and neurofascin (NFASC) (OR = 0.96, 95% CI = 0.92–1.00, *p* = 0.042). Initial findings for ITIH1 and ITIH3 were deemed unreliable due to pleiotropy (ITIH1: MR–Egger intercept *p* = 0.025) or heterogeneity (ITIH3: Cochran's *Q p* = 0.001). Furthermore, the MR analyses failed to yield evidence supporting a causal effect of liability to BD on neurodevelopment‐related proteins.

**Conclusion:**

The MR analysis indicated potential causal relationships between two neurodevelopment‐related proteins (NFASC and ITIH5) and BD. Further studies are required to validate these results and elucidate the specific functions of these proteins in the development of this mental disorder.

## Graphical abstract

The Mendelian randomization (MR) analysis investigating causal relationships between neurodevelopment‐related proteins and bipolar disorder (BD) is summarized here. Genetic instruments (SNPs) were used to assess effects. Results highlight ITIH5 as a risk factor (red arrow) and NFASC as a protective factor (green arrow) for BD, supporting the neurodevelopmental hypothesis of BD.

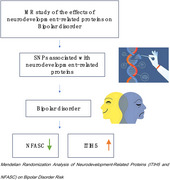

**Abbreviations**
BD: bipolar disorder
GWAS: genome‐wide association study
IVW: inverse‐variance weighted
MR: Mendelian randomization
PGC: Psychiatric Genomics Consortium
SNP: single nucleotide polymorphism

## 1 Introduction

Bipolar disorder (BD) is a debilitating psychiatric condition characterized by recurrent episodes of mania and depression, affecting approximately 2% of the global population (Merikangas et al. 2007). Despite its high heritability and substantial socioeconomic burden, the biological mechanisms underlying BD remain poorly understood. The neurodevelopmental hypothesis posits that disruptions in early brain maturation contribute to BD pathogenesis, supported by overlapping genetic and pathophysiological features with schizophrenia (SCZ) (Insel, 2010; Rund, 2018). For instance, both disorders share genetic risk loci (e.g., CACNA1C, ITIH3) and exhibit prodromal neurocognitive deficits (Cannon et al. 2015; Cross‐Disorder Group of the Psychiatric Genomics Consortium 2013). However, robust evidence directly linking neurodevelopmental pathways to BD is limited, and the disorder is not classified as neurodevelopmental in current diagnostic frameworks (First, 2013). Identifying specific neurodevelopmental factors causal to BD could transform diagnostic and therapeutic strategies.

Proteins critical to neurodevelopment are compelling candidates for elucidating this link. Genome‐wide association studies (GWASs) have implicated genes encoding neurodevelopment‐related proteins in BD and SCZ, suggesting shared etiological pathways (Stahl et al. 2019; Schizophrenia Working Group of the Psychiatric Genomics Consortium 2014). For example: The ITIH family (ITIH1–ITIH5): This gene cluster on chromosome 3p21 regulates extracellular matrix stability and neuroinflammation. Variants in ITIH1–ITIH4 are associated with SCZ and major depressive disorder (Li et al. 2017; Muglia et al. 2010). Despite these links, the role of ITIH proteins in BD remains underexplored. Neurofascin (NFASC): Essential for axon guidance and node of Ranvier formation, NFASC mutations cause neurodevelopmental disorders with central and peripheral demyelination (Gao et al. 2021). A sex‐specific association between NFASC rs2802808 and SCZ further underscores its relevance to psychiatric pathophysiology (Gui et al. 2022). These proteins were selected based on their established roles in neurodevelopmental processes and prior genetic associations with psychiatric disorders. However, causal relationships between these proteins and BD remain untested.

Mendelian randomization (MR) offers a powerful approach to address this gap by leveraging genetic variants as instrumental variables, minimizing confounding and reverse causation (Gui et al. 2022). Here, we performed a bidirectional MR analysis to evaluate potential causal effects between six neurodevelopment‐related proteins (ITIH1–ITIH5, NFASC) and BD. Our findings implicate ITIH5 and NFASC in BD etiology, providing novel insights into the neurodevelopmental origins of this complex disorder.

## 2 Materials and Methods

### 2.1 Study Design

This study aimed to determine the causal relationships between neurodevelopment‐related proteins and BD using a bidirectional MR approach. A robust MR study must meet three fundamental assumptions: (1) genetic variants should exhibit a strong association with the exposure; (2) genetic variants should be independent of any confounding variables that may exist between the exposure and the outcome; and (3) genetic variants that are significantly associated with the exposure should not have a direct effect on the outcome; that is, their influence on the outcome should occur exclusively through the exposure (Davey Smith and Hemani 2014; Lawlor 2016).

### 2.2 Data Sources for Exposure

Genetic variants on neurodevelopment‐related protein levels (pQTLs) were obtained from an extensive GWAS with 5368 European descents performed by Gudjonsson et al. (Lawlor et al. 2008). A total of 5368 European descents were included, and approximately 4035 single nucleotide polymorphisms (SNPs) were tested in this study. These data are publicly available from the GWAS Catalog (accession numbers: GCST90089947, GCST90090631, GCST90089692, GCST90088776, GCST90090066, and GCST90089710). In total, six neurodevelopment‐related proteins were included in the analysis: ITIH1, ITIH2, ITIH3, ITIH4, ITIH5, and NFASC.

### 2.3 Data Sources for the Outcome

Genetic variants for BD were obtained from the GWAS of the Psychiatric Genomics Consortium (PGC). The GWAS meta‐analysis included 57 BD cohorts of European ancestry, collected from North America, Europe, and Australia, with a total sample size of 413,466 individuals (*N*_Case_ = 41,917, *N*_Control_ = 371,549). The BD GWAS included mixed subtypes (I/II). Demographic details (e.g., 58% female, mean age = 42 years) are described in the PGC study (Gudjonsson et al. 2022). These data are publicly accessible on the PGC website (https://www.med.unc.edu/pgc/results‐and‐downloads).

Written informed consent had already been obtained from the participants of those GWASs, and the authors of those studies had received approval from the respective ethics committees. As our study used publicly available summary‐level data, no additional ethical approval was required.

### 2.4 Selection of Genetic Variants

In accordance with the three above‐mentioned fundamental assumptions of an MR study, the following steps were executed. First, in the forward MR analysis, SNPs significantly associated with neurodevelopment‐related proteins were identified using a significance threshold of *p* less than 5 × 10^−8^. Owing to the limited number of SNPs detected for neurodevelopment‐related proteins in general, we referred to previous MR studies on psychiatric disorders and applied a relatively lenient significance threshold (*p* < 5 × 10^−7^). Additionally, an even more lenient threshold (*p* < 5 × 10^−6^) was applied for certain proteins, such as NFASC. For the reverse MR analysis, a significance threshold of *p* less than 5 × 10^−8^ was used to identify SNPs associated with BD. To avoid bias, the parameters (kb = 10,000, *r* ^2^ = 0.001) were set to eliminate linkage disequilibrium and ensure the independence of the selected SNPs. Second, to satisfy the assumptions of independence and exclusivity, each SNP was queried on the PhenoScanner V2 database, and those linked to confounding factors (e.g., smoking behavior, alcohol consumption, socioeconomic status, and educational attainment) and BD were manually excluded. This ensured that the selected SNPs were independent of potential confounders between the outcome and exposure and had no direct relationship with the outcome, influencing it only through the exposure. Finally, we aligned the exposure and outcome SNPs to ensure consistent effect estimates for the same effect allele and excluded palindromic SNPs.

### 2.5 Mendelian Randomization Analyses

**FIGURE 1:**
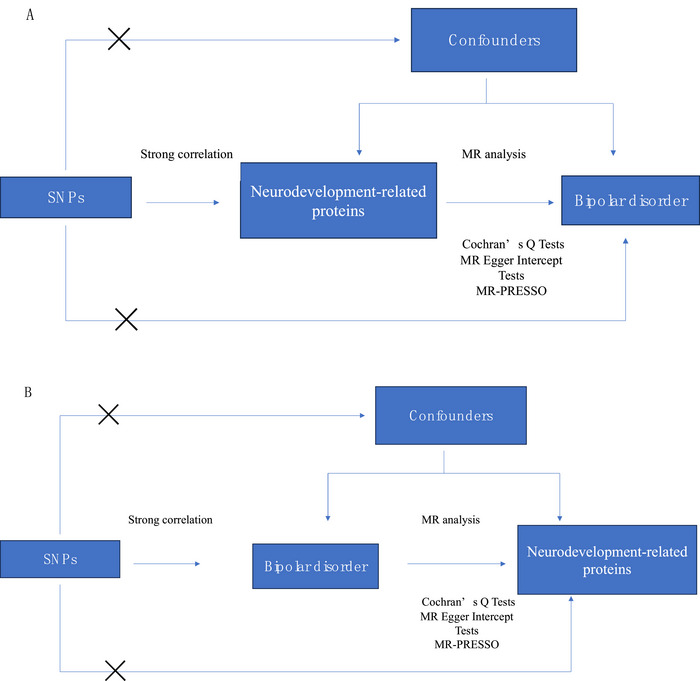
(A) MR analysis of neurodevelopment‐related proteins and bipolar disorder. (B) MR analysis of bipolar disorder and neurodevelopment‐related proteins.

The inverse‐variance weighted (IVW) approach (Mullins et al. 2021) was used as the principal analytical method to assess the causal relationship between neurodevelopment‐related proteins and BD. The simple mode (Burgess et al. 2013), weighted mode (Zhu et al. 2022), weighted median (Hartwig et al. 2017), and MR–Egger methods (Bowden et al. 2016) were used for additional analyses. Heterogeneity was quantified using Cochran's *Q* test, where a *p* value of less than 0.05 denotes significant heterogeneity (Bowden et al. 2015). Horizontal pleiotropy was evaluated using MR–Egger regression (MR–Egger intercept test), with a *p* value greater than 0.05 indicating no evidence of such effect (Sterne et al. 2011). The MR pleiotropy residual sum and outlier (MR‐PRESSO) test was used to identify and remove horizontal pleiotropic outliers. Associations between neurodevelopment‐related proteins and BD were presented as odds ratios (ORs) with 95% confidence intervals (CIs). The MR analyses were performed using the MR‐PRESSO and TwoSampleMR packages in R (version 4.3.1). The study profile is shown in Figure 1.

## 3 Results

### 3.1 Mendelian Randomization of the Effect of Neurodevelopment‐Related Proteins on Bipolar Disorder

**FIGURE 2:**
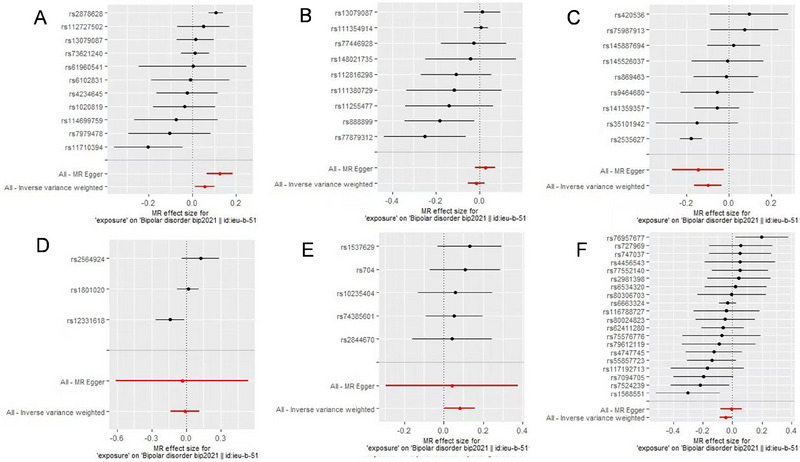
Causal effects of neurodevelopment‐related proteins on bipolar disorder. In this Mendelian randomization study, neurodevelopment‐related proteins were analyzed as the exposure factor, with bipolar disorder as the resultant outcome. (A) ITIH1, (B) ITIH2, (C) ITIH3, (D) ITIH4, (E) ITIH5, and (F) NFASC.

**FIGURE 3:**
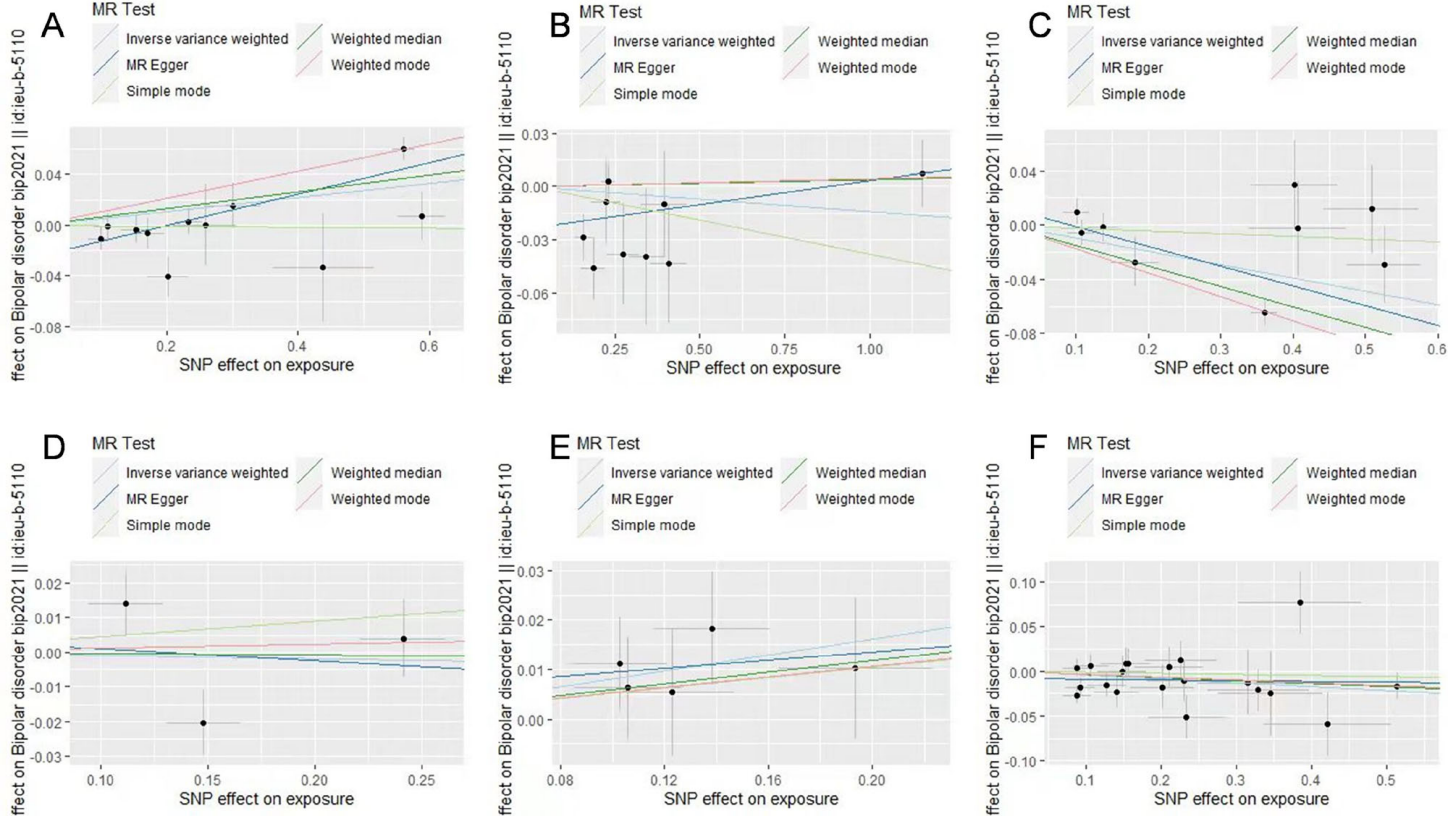
Scatter plots of the associations between neurodevelopment‐related proteins and bipolar disorder. Different Mendelian randomization methods are indicated by corresponding colored lines. (A) ITIH1, (B) ITIH2, (C) ITIH3, (D) ITIH4, (E) ITIH5, and (F) NFASC.

Table 1 presents the causal effects of neurodevelopment‐related proteins on BD based on the IVW, weighted median, weighted mode, MR–Egger, and simple mode methods. According to the IVW results, ITIH1 (OR = 1.06, 95% CI = 1.01–1.10, *p* = 0.013) and ITIH5 (OR = 1.08, 95% CI = 1.00–1.17, *p* = 0.04) were risk factors for BD, whereas ITIH3 (OR = 0.91, 95% CI = 0.85–0.97, *p* = 3.22e‐03) and NFASC (OR = 0.96, 95% CI = 0.92–1.00, *p* = 0.042) were protective factors. By contrast, the proteins ITIH2 and ITIH4 had no significant causal effects on BD according to all five analytical methods (Table 1). Furthermore, the results of the weighted median, MR–Egger, simple mode, and weighted mode methods were consistent with those of the IVW approach in terms of effect direction. The forest and scatter plots generated using the five MR methods further validated the stability of the results (Figures 2 and 3).

**TABLE 1:** The causal effect of neurodevelopment‐related proteins on bipolar disorder.

Outcome	Exposures	SNPs	Method	OR (95% C.I.)	*p* value
BD	ITIH1	11	inverse‐variance weighted	1.06 (1.01–1.10)	0.013
			MR–Egger	1.13 (1.07–1.20)	0.003
			weighted median	1.07 (1.03–1.11)	0.001
			weighted mode	1.11 (1.07–1.15)	0.000
			simple mode	1.00 (0.91–1.08)	0.938
	ITIH2	9	inverse‐variance weighted	0.99 (0.95–1.03)	0.477
			MR–Egger	1.03 (0.98–1.08)	0.303
			weighted median	1.00 (0.98–1.08)	0.803
			weighted mode	1.00 (0.97–1.04)	0.776
			simple mode	0.96 (0.86–1.07)	0.510
	ITIH3	9	inverse‐variance weighted	0.91 (0.85–0.97)	3.2e‐03
			MR–Egger	0.86 (0.76–0.98)	5.2e‐02
			weighted median	0.86 (0.81–0.92)	4.5e‐06
			weighted mode	0.84 (0.79–0.89)	2.9e‐04
			simple mode	0.98 (0.87–1.10)	7.5e‐01
	ITIH4	3	inverse‐variance weighted	0.99 (0.87–1.12)	0.884
			MR–Egger	0.97 (0.54–1.72)	0.926
			weighted median	1.00 (0.92–1.08)	0.915
			weighted mode	1.01 (0.90–1.31)	0.861
			simple mode	1.05 (0.92–1.19)	0.562
	ITIH5	5	inverse‐variance weighted	1.08 (1.00–1.17)	0.040
			MR–Egger	1.04 (0.74–1.46)	0.826
			weighted median	1.06 (0.97–1.17)	0.219
			weighted mode	1.05 (0.93–1.19)	0.441
			simple mode	1.05 (0.93–1.19)	0.445
	NFASC	20	inverse‐variance weighted	0.96 (0.92–1.00)	0.042
			MR–Egger	0.99 (0.92–1.07)	0.836
			weighted median	0.97 (0.92–1.02)	0.188
			weighted mode	0.97 (0.92–1.02)	0.260
			simple mode	0.99 (0.90–1.09)	0.817

Heterogeneity was assessed using Cochran's *Q* test, whereas pleiotropy was evaluated using the MR–Egger intercept test (Table 2). No heterogeneity was observed in the MR analyses of the causal relationships of NFASC (*Q* = 25.25, *p* = 0.153) and ITIH5 (*Q* = 0.8, *p* = 0.938) with BD. Conversely, heterogeneity was observed in the analyses for ITIH1 (*Q* = 31.21, *p* < 0.001) and ITIH3 (*Q* = 25.2, *p* = 0.001).

**TABLE 2:** Results for Cochran's *Q* tests for heterogeneity and MR–Egger intercept tests for horizontal pleiotropy for the association between neurodevelopment‐related proteins and bipolar disorder.

		MR–Egger		Cochran's *Q*		MR‐PRESSO
Exposures	Outcome	MR–Egger Intercept (SE)	*p* value	Cochran's *Q* (df)	*p* value	MR‐PRESSO Global Test *p* value
ITIH1	BD	−0.025	0.025	31.21	< 0.001	0.037
ITIH2		−0.023	0.057	15.66	0.047	0.245
ITIH3		0.013	0.395	25.20	0.001	0.039
ITIH4		0.004	0.943	7.10	0.029	NA
ITIH5		0.005	0.828	0.80	0.938	0.946
NFASC		−0.008	0.261	25.25	0.153	0.228

Based on the MR–Egger intercept test, no evidence of horizontal pleiotropy was found for the SNPs of NFASC (intercept = 0.008, *p* = 0.621), ITIH3 (intercept = 0.013, *p* = 0.395), and ITIH5 (intercept = 0.005, *p* = 0.828). However, evidence of horizontal pleiotropy was observed among the SNPs of ITIH1 (intercept = 0.025, *p* = 0.025). Additionally, the MR‐PRESSO global test revealed no horizontal pleiotropy outliers in the MR analyses of NFASC and ITIH5.

These findings indicate that BD has robust causal relationships with NFASC and ITIH5, whereas its relationships with ITIH3 require further validation and that with ITIH1 is unreliable.

### 3.2 Mendelian Randomization of the Effect of Bipolar Disorder on Neurodevelopment‐Related Proteins

The MR analyses examining the impact of liability to BD on neurodevelopment‐related proteins did not yield evidence supporting a causal relationship (Table 3).

**TABLE 3:** The causal effect of bipolar disorder on neurodevelopment‐related proteins.

Outcomes	SNPs	Method	Beta	*p* value
ITIH1	44	Inverse‐variance weighted	0.24	0.223
		MR–Egger	0.67	0.556
		Weighted median	0.08	0.267
		Weighted mode	0.08	0.550
		Simple mode	0.08	0.568
ITIH2	44	Inverse‐variance weighted	0.03	0.569
		MR–Egger	0.26	0.385
		Weighted median	0.11	0.125
		Weighted mode	0.17	0.281
		Simple mode	0.19	0.250
ITIH3	44	Inverse‐variance weighted	−0.08	0.553
		MR–Egger	0.04	0.955
		Weighted median	0.03	0.690
		Weighted mode	0.02	0.901
		Simple mode	−0.01	0.957
ITIH4	44	Inverse‐variance weighted	0.04	0.416
		MR–Egger	0.57	0.031
		Weighted median	0.04	0.538
		Weighted mode	0.01	0.907
		Simple mode	0.01	0.980
ITIH5	44	Inverse‐variance weighted	−0.08	0.093
		MR–Egger	−0.03	0.913
		Weighted median	−0.07	0.336
		Weighted mode	−0.13	0.399
		Simple mode	−0.13	0.389
NFASC	44	Inverse‐variance weighted	−0.01	0.970
		MR–Egger	0.10	0.740
		Weighted median	0.03	0.684
		Weighted mode	0.03	0.849
		Simple mode	−0.01	0.983

## 4 Discussion

This study represents the first MR analysis utilizing publicly available GWAS data to establish a potential causal relationship between neurodevelopment‐related proteins (ITIH5 and NFASC) and BD. Our findings demonstrate that elevated ITIH5 levels may confer increased BD risk (OR = 1.08, *p* = 0.04), whereas higher NFASC expression appears protective (OR = 0.96, *p* = 0.042). Notably, these associations remained robust after controlling for pleiotropy and heterogeneity, supporting their potential etiological relevance.

As a member of the inter‐alpha‐trypsin inhibitor family, ITIH5 regulates extracellular matrix stability and neuroinflammatory responses (Burgess and Thompson 2017). Prior proteomic studies have documented elevated ITIH5 levels in SCZ patients compared to healthy controls (Sessler et al. 2024), suggesting shared pathophysiological mechanisms across psychiatric disorders. NFASC, critical for axon guidance and node of Ranvier formation (Wang et al. 2022; Ghosh et al. 2018; Song et al. 2023), has been implicated in neurodevelopmental disorders with peripheral demyelination (Dong et al. 2022). The observed inverse association between NFASC and BD aligns with emerging evidence linking axonal integrity deficits to mood dysregulation (Efthymiou et al. 2019; Sherman et al. 2005).

While our analysis initially suggested a protective effect of ITIH3 against BD (IVW OR = 0.91, *p* = 3.22e‐03), significant heterogeneity among SNPs (Cochran's *Q p* = 0.001) necessitates cautious interpretation. This parallels prior GWAS findings where ITIH3 polymorphisms exhibited pleiotropic associations with both SCZ and BD across populations (Buttermore et al. 2012; Takeuchi et al. 2022; Hamshere et al. 2013). Similarly, the putative risk effect of ITIH1 (IVW OR = 1.06, *p* = 0.013) was compromised by detectable horizontal pleiotropy (MR–Egger intercept *p* = 0.025), echoing inconsistencies in earlier MR studies (Psychiatric GWAS Consortium Bipolar Disorder Working Group 2011; Hu et al. 2023). These discrepancies underscore the need for larger‐scale proteomic GWAS to clarify causal relationships.

The bidirectional MR design enhances causal inference by minimizing confounding from environmental factors and reverse causation. The use of independent exposure/outcome datasets (European ancestry) improved statistical power while reducing population stratification bias. However, several limitations warrant attention: While our analysis incorporated data from diverse BD cohorts to mitigate potential ethical biases, a critical limitation lies in the restricted ancestral diversity of the samples. The GWAS datasets for both neurodevelopment‐related proteins and BD were predominantly derived from European populations. This homogeneity precludes extrapolation of our findings to other ethnic groups, particularly given known interethnic differences in ITIH allele frequencies (Dang et al. 2023). MR inherently detects linear relationships, whereas protein‐disease associations may follow nonlinear thresholds. Unadjusted sex/age stratification analyses obscure potential modifiers, as NFASC exhibits sex‐dimorphic expression in neurodevelopmental pathways (Xie et al. 2020). Validation in multi‐ancestry cohorts and integration with longitudinal proteomic profiling could resolve current ambiguities. Mechanistic studies exploring ITIH5/NFASC interactions with synaptic pruning, myelination, and neuroimmune pathways may elucidate their roles in BD pathogenesis. Additionally, transcriptome‐wide MR analyses could identify upstream regulatory networks linking these proteins to clinical phenotypes.

## 5 Conclusions

Our findings suggest potential causal relationships between NFASC and ITIH5 and BD. This discovery provides novel insights into the pathophysiology of BD and additional evidence to support the neurodevelopmental hypothesis of the disorder. Further investigation into the specific roles of these neurodevelopment‐related proteins in the pathophysiology of BD is warranted.

## Author Contributions

**Yanyan Li**: writing–original draft, formal analysis. **Qianqian Gui**: writing–original draft, formal analysis. **Shurong Ren**: writing–original draft, formal analysis. **Zhifen Liu**: writing–review and editing. **Aixia Zhang**: writing–review and editing. **Penghong Liu**: writing–review and editing. **Xueping Zhou**: writing–review and editing. **Ning Sun**: conceptualization, supervision. **Chunxia Yang**: conceptualization, supervision.

## Consent

The authors have nothing to report.

## Conflicts of Interest

The authors declare no conflicts of interest.

### Peer Review

The peer review history for this article is available at https://publons.com/publon/10.1002/brb3.70442

## Data Availability

All data used for this study are publicly available.

## References

[brb370442-bib-0002] Bowden, J. , G. Davey Smith , and S. Burgess . 2015. “Mendelian Randomization With Invalid Instruments: Effect Estimation and Bias Detection Through Egger Regression.” International Journal of Epidemiology 44: 512–525. 26050253 10.1093/ije/dyv080 PMC4469799

[brb370442-bib-0003] Bowden, J. , G. Davey Smith , P. C. Haycock , and S. Burgess . 2016. “Consistent Estimation in Mendelian Randomization With Some Invalid Instruments Using a Weighted Median Estimator.” Genetic Epidemiology 40: 304–314. 27061298 10.1002/gepi.21965 PMC4849733

[brb370442-bib-0004] Burgess, S. , A. Butterworth , and S. G. Thompson . 2013. “Mendelian Randomization Analysis With Multiple Genetic Variants Using Summarized Data.” Genetic Epidemiology 37: 658–665. 24114802 10.1002/gepi.21758 PMC4377079

[brb370442-bib-0005] Burgess, S. , and S. G Thompson . 2017. “Interpreting Findings From Mendelian Randomization Using the MR‐Egger Method.” European Journal of Epidemiology 32: 377–389. https://pubmed.ncbi.nlm.nih.gov/28527048/. 28527048 10.1007/s10654-017-0255-x PMC5506233

[brb370442-bib-0006] Buttermore, E. D. , C. Piochon , M. L. Wallace , B. D. Philpot , C. Hansel , and M. A. Bhat . 2012. “Pinceau Organization in the Cerebellum Requires Distinct Functions of Neurofascin in Purkinje and Basket Neurons During Postnatal Development.” Journal of Neuroscience 32: 4724–4742. 22492029 10.1523/JNEUROSCI.5602-11.2012 PMC3337041

[brb370442-bib-0007] Cannon, T. D. , Y. Chung , G. He , et al. 2015. “Progressive Reduction in Cortical Thickness as Psychosis Develops: A Multisite Longitudinal Neuroimaging Study of Youth at Elevated Clinical Risk.” Biological Psychiatry 77: 147–157. 25034946 10.1016/j.biopsych.2014.05.023 PMC4264996

[brb370442-bib-0008] Cross‐Disorder Group of the Psychiatric Genomics Consortium . Hong Lee, S. , S. Ripke , et al. 2013. “Genetic Relationship Between Five Psychiatric Disorders Estimated From Genome‐Wide SNPs.” Nature Genetics 45: 984–994. 23933821 10.1038/ng.2711 PMC3800159

[brb370442-bib-0009] Dang, X. , M. Song , L. Lv , Y. Yang , and X. J. Luo . 2023. “Proteome‐Wide Mendelian Randomization Reveals the Causal Effects of Immune‐Related Plasma Proteins on Psychiatric Disorders.” Human Genetics 142: 809–818. 37085628 10.1007/s00439-023-02562-0

[brb370442-bib-0010] Davey Smith, G. , and G. Hemani . 2014. “Mendelian Randomization: Genetic Anchors for Causal Inference in Epidemiological Studies.” Human Molecular Genetics 23: R89–R98. 25064373 10.1093/hmg/ddu328 PMC4170722

[brb370442-bib-0011] Dong, Y. , Y. Chen , B. Yao , et al. 2022. “Neuropathologic Damage Induced by Radiofrequency Ablation at Different Temperatures.” Clinics 77: 100033. 35436702 10.1016/j.clinsp.2022.100033 PMC9035646

[brb370442-bib-0012] Efthymiou, S. , V. Salpietro , N. Malintan , et al. 2019. “Biallelic Mutations in Neurofascin Cause Neurodevelopmental Impairment and Peripheral Demyelination.” Brain 142: 2948–2964. 31501903 10.1093/brain/awz248 PMC6763744

[brb370442-bib-0013] First, M. B. 2013. “Diagnostic and Statistical Manual of Mental Disorders, 5th Edition, and Clinical Utility.” Journal of Nervous and Mental Disease 201: 727–729. 23995026 10.1097/NMD.0b013e3182a2168a

[brb370442-bib-0014] Gao, Y. , L. Kong , S. Liu , K. Liu , and J. Zhu . 2021. “Impact of Neurofascin on Chronic Inflammatory Demyelinating Polyneuropathy via Changing the Node of Ranvier Function: A Review.” Frontiers in Molecular Neuroscience 14: 779385. 34975399 10.3389/fnmol.2021.779385 PMC8716720

[brb370442-bib-0015] Ghosh, A. , D. L. Sherman , and P. J. Brophy . 2018. “The Axonal Cytoskeleton and the Assembly of Nodes of Ranvier.” Neuroscientist 24: 104–110. 28534438 10.1177/1073858417710897 PMC5846858

[brb370442-bib-0016] Gudjonsson, A. , V. Gudmundsdottir , G. T. Axelsson , et al. 2022. “A Genome‐Wide Association Study of Serum Proteins Reveals Shared Loci With Common Diseases.” Nature Communications 13: 480. 10.1038/s41467-021-27850-z PMC8789779 35078996

[brb370442-bib-0017] Gui, Y. , X. Zhou , Z. Wang , et al. 2022. “Sex‐Specific Genetic Association Between Psychiatric Disorders and Cognition, Behavior and Brain Imaging in Children and Adults.” Translational Psychiatry 12: 347. 36028495 10.1038/s41398-022-02041-6 PMC9418275

[brb370442-bib-0018] Hamshere, M. L. , J. T. R. Walters , R. Smith , et al. 2013. “Genome‐Wide Significant Associations in Schizophrenia to ITIH3/4, CACNA1C and SDCCAG8, and Extensive Replication of Associations Reported by the Schizophrenia PGC.” Molecular Psychiatry 18: 708–712. 22614287 10.1038/mp.2012.67 PMC4724864

[brb370442-bib-0019] Hartwig, F. P. , G. Davey Smith , and J. Bowden . 2017. “Robust Inference in Summary Data Mendelian Randomization via the Zero Modal Pleiotropy Assumption.” International Journal of Epidemiology 46: 1985–1998. 29040600 10.1093/ije/dyx102 PMC5837715

[brb370442-bib-0020] Hu, J. J. , Y. B. Zhang , S. F. Zheng , et al. 2023. “The Causal Relationship Between Circulating Biomarkersand the Risk of Bipolar Disorder: A Two‐Sample Mendelian Randomization Study.” Journal of Psychiatric Research 164: 66–71. 37327502 10.1016/j.jpsychires.2023.05.070

[brb370442-bib-0021] Insel, T. R. 2010. “Rethinking Schizophrenia.” Nature 468: 187–193. https://pubmed.ncbi.nlm.nih.gov/21068826/. 21068826 10.1038/nature09552

[brb370442-bib-0022] Lawlor, D. A. 2016. “Commentary: Two‐Sample Mendelian Randomization: Opportunities and Challenges.” International Journal of Epidemiology 45: 908–915. 27427429 10.1093/ije/dyw127 PMC5005949

[brb370442-bib-0023] Lawlor, D. A. , R. M. Harbord , J. A. C. Sterne , N. Timpson , and G. Davey Smith . 2008. “Mendelian Randomization: Using Genes as Instruments for Making Causal Inferences in Epidemiology.” Statistics in Medicine 27: 1133–1163. 17886233 10.1002/sim.3034

[brb370442-bib-0024] Li, Z. , J. Chen , H. Yu , et al. 2017. “Genome‐Wide Association Analysis Identifies 30 New Susceptibility Loci for Schizophrenia.” Nature Genetics 49: 1576–1583. 28991256 10.1038/ng.3973

[brb370442-bib-0025] Merikangas, K. R. , H. S. Akiskal , J. Angst , et al. 2007. “Lifetime and 12‐Month Prevalence of Bipolar Spectrum Disorder in the National Comorbidity Survey Replication.” Archives of General Psychiatry 64: 543–552. 17485606 10.1001/archpsyc.64.5.543 PMC1931566

[brb370442-bib-0026] Muglia, P. , F. Tozzi , N. W. Galwey , et al. 2010. “Genome‐Wide Association Study of Recurrent Major Depressive Disorder in Two European Case‐Control Cohorts.” Molecular Psychiatry 15: 589–601. 19107115 10.1038/mp.2008.131

[brb370442-bib-0027] Mullins, N. , A. J. Forstner , K. S. O'connell , et al. 2021. “Genome‐Wide Association Study of More Than 40,000 Bipolar Disorder Cases Provides New Insights Into the Underlying Biology.” Nature Genetics 53: 817–829. 34002096 10.1038/s41588-021-00857-4 PMC8192451

[brb370442-bib-0028] Psychiatric GWAS Consortium Bipolar Disorder Working Group . 2011. “Large‐Scale Genome‐Wide Association Analysis of Bipolar Disorder Identifies a New Susceptibility Locus Near ODZ4.” Nature Genetics 43: 977–983. 21926972 10.1038/ng.943 PMC3637176

[brb370442-bib-0029] Rund, B. R. 2018. “The Research Evidence for Schizophrenia as a Neurodevelopmental Disorder.” Scandinavian Journal of Psychology 59: 49–58. 29356007 10.1111/sjop.12414

[brb370442-bib-0030] Schizophrenia Working Group of the Psychiatric Genomics Consortium . 2014. “Biological Insights From 108 Schizophrenia‐Associated Genetic Loci.” Nature 511: 421–427. https://pubmed.ncbi.nlm.nih.gov/25056061/. 25056061 10.1038/nature13595 PMC4112379

[brb370442-bib-0031] Sessler, T. M. , J. P. Beier , S. Villwock , D. Jonigk , E. Dahl , and T. Ruhl . 2024. “Genetic Deletion of ITIH5 Leads to Increased Development of Adipose Tissue in Mice.” Biological Research 57: 58. 39198923 10.1186/s40659-024-00530-0 PMC11360682

[brb370442-bib-0032] Sherman, D. L. , S. Tait , S. Melrose , et al. 2005. “Neurofascins Are Required to Establish Axonal Domains for Saltatory Conduction.” Neuron 48: 737–742. 16337912 10.1016/j.neuron.2005.10.019

[brb370442-bib-0033] Song, Z. , Z. Wu , R. Luo , et al. 2023. “Identification of Tryptophan Metabolism‐Related Genes in Immunity and Immunotherapy in Alzheimer's Disease.” Aging 15: 13077–13099. 37988184 10.18632/aging.205220 PMC10713402

[brb370442-bib-0034] Stahl, E. A. , G. Breen , A. J. Forstner , et al. 2019. “Genome‐Wide Association Study Identifies 30 Loci Associated With Bipolar Disorder.” Nature Genetics 51: 793–803. 31043756 10.1038/s41588-019-0397-8 PMC6956732

[brb370442-bib-0035] Sterne, J. A. C. , A. J. Sutton , J. P. A. Ioannidis , et al. 2011. “Recommendations for Examining and Interpreting Funnel Plot Asymmetry in Meta‐Analyses of Randomised Controlled Trials.” BMJ 343: d4002. 21784880 10.1136/bmj.d4002

[brb370442-bib-0036] Takeuchi, H. , H. Tomita , Y. Taki , et al. 2022. “A Psychiatric Disorder Risk Polymorphism of ITIH3 Is Associated With Multiple Neuroimaging Phenotypes in Young Healthy Adults.” Psychiatry and Clinical Neurosciences 76: 271–273. 35261121 10.1111/pcn.13347

[brb370442-bib-0037] Wang, L. J. , Y. C. Huang , P. Y. Lin , et al. 2022. “BST‐1 as a Serum Protein Biomarker Involved in Neutrophil Infiltration in Schizophrenia.” World Journal of Biological Psychiatry 23: 537–547. 10.1080/15622975.2021.2014151 34870552

[brb370442-bib-0038] Xie, X. , H. Meng , H. Wu , et al. 2020. “Integrative Analyses Indicate an Association Between ITIH3 Polymorphisms With Autism Spectrum Disorder.” Scientific Reports 10: 5223. 32251353 10.1038/s41598-020-62189-3 PMC7089985

[brb370442-bib-0039] Zhu, G. , S. Zhou , Y. Xu , et al. 2022. “Mendelian Randomization Study on the Causal Effects of Omega‐3 Fatty Acids on Rheumatoid Arthritis.” Clinical Rheumatology 41: 1305–1312. 35000008 10.1007/s10067-022-06052-y

